# Research progress of online monitoring techniques for dissolved gas analysis in insulating oil

**DOI:** 10.3389/fchem.2025.1721893

**Published:** 2025-12-17

**Authors:** Hua Mao, Fangqiang Wang, Jie Wang, Zeyao Ma, Yan Wang, Rui Peng, Zhi Geng, Xinsheng Lan

**Affiliations:** 1 State Grid Sichuan Electric Power Research Institute, Chengdu, Sichuan, China; 2 Power System Security and Operation Key Laboratory of Sichuan Province, Chengdu, Sichuan, China; 3 Sichuan Shuneng Electric Power Co., Ltd., Chengdu, Sichuan, China

**Keywords:** dissolved gas analysis, fault diagnosis, insulating oil, online monitoring, transformer

## Abstract

Dissolved gas analysis (DGA) in insulating oil can achieve early warning of local discharge, overheating or arc failure in transformers. It can also quantitatively evaluate the aging degree of insulating equipment, providing a basis for formulating predictive maintenance strategies, so as to improve the reliability of power grid power supply. At present, the existing common offline detection methods take a long time, and the dissolved gases in transformers are usually flammable and explosive, posing potential hazards to on-site maintenance personnel. Therefore, it is urgent to develop online detection methods for DGA. This article introduces the types and milestone research progress of DGA online detection methods in recent years (2019–2025). The instant sensing methods of dissolved gas in transformer oil are summarized and analyzed, including on-line gas chromatography analysis system, spectral analysis, and gas sensor methods. Through summarizing the advantages of different methods in terms of detection limit, response speed, operation and maintenance cost, etc., it puts forward the development trend of promoting intelligent and accurate power equipment status monitoring. This review aims to promote the further development of on-line DGA and contribute to the construction of an intelligent grid protection system by providing important technical insights.

## 1 Introduction

Insulating oils are used in power transformers as dielectric fluids with good physical-chemical properties, and in addition to insulating, this oil dissipates heat generated in the magnetic circuit and windings. Mineral insulating oils in transformers provide dielectric strength, effective cooling, protection of the transformer core and coil assemblies from chemical intrusion, and prevention of sludge buildup (Simões et al., 2024). The insulating oil, as it moves through the operating transformer, conducts heat to the internal cooling system (radiators, etc.) before releasing it to the environment (Guerbas et al., 2024). More than 90% of transformers are oil-immersed power transformers, (Ali et al., 2023), Transformer oil, whose primary component is mineral oil, constitutes a complex hydrocarbon mixture dominated by alkanes, naphthenes, aromatic hydrocarbons, and functional additives (Cui et al., 2019). When electrical and thermal defects occur in transformer oils, they degrade the resulting combustible gases such as hydrogen (H_2_), ethylene (C_2_H_4_), acetylene (C_2_H_2_), methane (CH_4_) and ethane (C_2_H_6_). When cellulose insulation decomposes, the gases produced are carbon monoxide (CO) and carbon dioxide (CO_2_) (Bustamante et al., 2019). In addition to this, faults such as conductor loss, heat from arcing, partial discharges from ion bombardment, etc., all produce different gases. (Faiz and Soleimani, 2017). These gases not only reflect the fault problems of transformers, but more importantly, these flammable and explosive gases pose significant safety hazards to the operation and maintenance of power equipment. Therefore, timely detection and analysis of these dissolved gases have significant practical significance.

Dissolved gas analysis (DGA) in transformer oil is a core technology for diagnosing potential faults in power equipment and plays an important role in the industry (Mahmoudi et al., 2019). DGA relates the concentration of various insulation degradation by-products dissolved in the oil to the nature of the fault, and can be used to identify initial faults and monitor the operating conditions of oil-immersed transformers (Wani et al., 2021), assessing the degree of deterioration and providing a basis for developing predictive maintenance strategies (Bustamante et al., 2024). Current mainstream detection techniques include gas chromatography (GC), gas chromatography-mass (GC-MS). These technologies require collecting samples of dissolved gases in insulation oil in transformers, and then analyzing and testing them using large-scale gas chromatography instruments in the laboratory. Although offline gas chromatography technology has high accuracy in detecting dissolved gases, there are many problems: it requires offline collection of insulation oil samples, which not only increases the complexity of the detection process, but also poses a certain personal danger to power equipment maintenance personnel; It is time-consuming, cumbersome to operate, and inefficient, and cannot achieve real-time, intuitive, and effective monitoring of power transformers; It is difficult to meet the real-time monitoring needs of power transformers in operation, which in turn affects the stability of equipment operation and the accuracy of testing. Therefore, it is urgent to develop online monitoring technology for dissolved gases in insulating oil.

**FIGURE 1:**
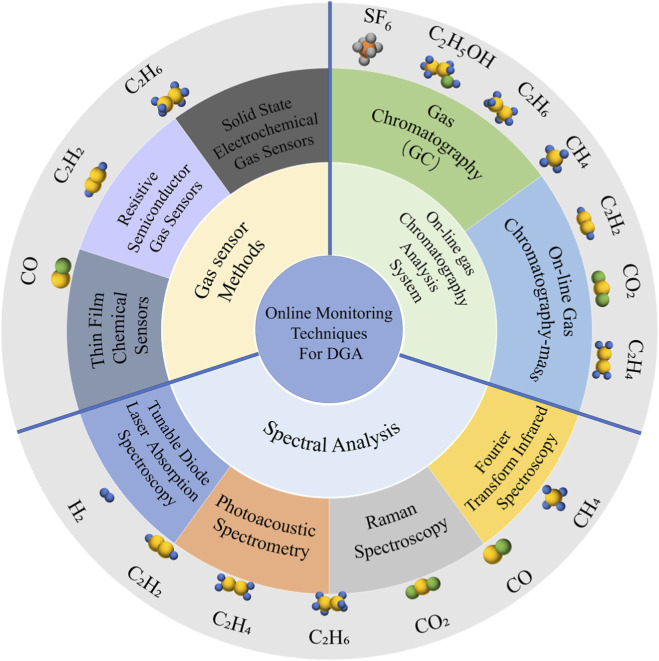
Schematic diagram summarizing the main contents of this review.

Online-monitoring technology refers to sensing devices and data acquisition systems that can obtain and analyze real-time dynamic parameters of dissolved gases in oil while power equipment is continuously operating. The sensitivity of online sensing and detection methods directly affects the accuracy of monitoring technology. As shown in Figure 1, online monitoring technology includes online gas chromatography analysis system (online gas chromatography, online gas chromatography-mass), spectral analysis (Fourier transform infrared spectroscopy (FTIR), Raman spectroscopy (RS), photoacoustic spectroscopy (PAS), tunable diode laser absorption spectroscopy (TDLAS)) and gas sensors (ethane sensor, carbon monoxide sensor, acetylene sensor). As shown in Table 1, the different methods have their own advantages in terms of detection limits, response speed, and operation and maintenance costs, and have driven the development of intelligent and accurate monitoring of power equipment condition (Ayala-Cabrera et al., 2022). Online-monitoring methods of DGA are becoming the development direction with high potential. This review revolves around three core objectives: 1. Systematically classify and compare the operating principles and performance indicators (such as detection limits, response speed, and multi gas capability) of mainstream online DGA technologies developed in the past 5 years (2019–2025). 2. Critically analyze the key challenges and bottlenecks that hinder the widespread industrial application of these technologies. 3. Based on comparative analysis, provide a forward-looking perspective on the most promising research directions and technological trends, which may lead to the development of more intelligent, accurate, and economically feasible monitoring solutions for the power industry.

**TABLE 1:** Comparative analysis of online DGA techniques.

Detection techniques	Advantage	Disadvantage
GC	Powerful separation capability, good stability, fast analysis speed, high degree of automation, and high sensitivity	Easy to be affected by cross sensitivity, columns are prone to aging, replacement is time-consuming and costly
GC-MS	High sensitivity, low detection limit, high precision	High equipment cost and complex operation
FTIR	Fast detection speed	Limitations (inability to detect H_2_), susceptibility to environmental influences, large instrument size, high maintenance costs
RS	Non destructive testing, rapid analysis, simplicity, no need to separate gas mixtures, stability testing	Low sensitivity
PAS	Strong anti-interference ability, fast response time, compact structure, high sensitivity, non-destructive testing, dynamic range, low cost, small footprint	System complexity, high cost, and high maintenance requirements
TDLAS	Good selectivity, high sensitivity, fast response time, and high anti-interference ability	System complexity and high cost
Gas sensors	High sensitivity, fast response, miniaturization and integration, no need for complex degassing	Lack of selectivity, instability, and frequent calibration due to long-term oil immersion causing material degradation

## 2 On-line gas chromatography analysis system

### 2.1 On-line gas chromatography

On-line gas chromatography is a method for separating and analyzing gas mixtures based on the affinity and adsorption capacity of different substances through direct sampling and real-time measurements. It is a relatively simple and mature technique, and its core advantage is its strong separation ability, which enables it to efficiently differentiate components in complex mixtures. In addition, the technique is characterized by good stability, fast analysis speed, high degree of automation, and excellent sensitivity, which enables real-time data monitoring, provides timely and reliable analysis results, and significantly improves the accuracy and efficiency of process control (Ayala-Cabrera et al., 2022). However, columns are susceptible to aging (Chen et al., 2019). It has a large device size and is dependent on the carrier gas and column (Ma et al., 2017), as well as the fact that the column is easily contaminated and the whole process of replacing the column is complicated, make the method time-consuming and costly (Mao et al., 2025; Tian et al., 2023; Wang Y. et al., 2020; Wang et al., 2021). More so, physical or chemical detectors are susceptible to cross-sensitivity, while electromagnetic interference and reliability can also affect detection accuracy (Wang et al., 2018).

**FIGURE 2:**
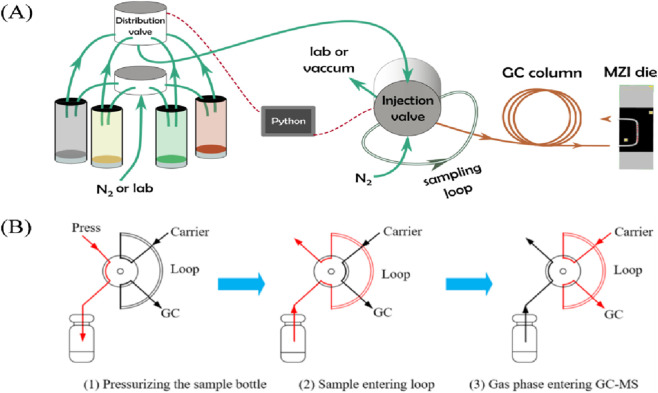
**(A)** Gas flow schematics of the chromatographic setup (Paris et al., 2024). **(B)** Sample cycle operating mode (Zheng et al., 2020).

In the past 5 years, researchers have carried out some improvement studies on on-line gas chromatography. For example, in 2023, Zhang et al. used a gas chromatography online monitoring device to carry out fault analysis. The core of the device consists of a degassing module and a detection module, and the detection module adopts a dual-column-detector structure: column 1 separates CO_2_, C_2_H_4_, C_2_H_6_, C_2_H_2_, and the detection is accomplished by detector 1 (based on the Wheatstone bridge principle); column 2 separates H_2_, CO, and CH_4_ and the detection is accomplished by detector 2. The malfunction was investigated through the steps of hardware status verification, data comparison, and taking cross experiments. After repairing, the results show that the absolute and relative errors between the detection value of the online device and the offline chromatographic data in the laboratory satisfy the Q/GDW10536-2021 standard (Zhang et al., 2023). In 2024, Sheng (2024) developed an online monitoring system for dissolved gases in transformer oil using vacuum pressure difference degassing and gas chromatography separation technology. The system can effectively separate and detect fault characteristic gases such as H_2_, CO, CO_2_, and hydrocarbons, with a minimum detection limit of 5.0 ppm for H_2_. (Sheng, 2024) In the same year, Pomme Hirschauer et al. reported a novel integrated photonic platform for analytical detection in gas chromatography. This work opened up new strategies for fast, low-cost, low detection limits for specific gas chromatography silicon microdetectors. Figure 2A is a description of the Gas flow schematics of the chromatographic setup (Paris et al., 2024).

GC is currently the most widely used and technologically mature online DGA solution in industrial sites, and has been adopted as a standard configuration by many power companies worldwide. The test results are consistent with the offline laboratory benchmark method and have recognized authority. In the future industrial development, the focus should be on further reducing its maintenance complexity and long-term operating costs through modular design, chromatographic column life prediction, and automatic calibration algorithms, in order to consolidate its core position in key transformer monitoring.

### 2.2 On-line gas chromatography-mass

Gas chromatography-mass spectrometry (GC-MS) is an on-line monitoring method that connects gas chromatography and mass spectrometry. The working principle of GC-MS is: the sample is first separated by gas chromatography (GC), and each component is sequentially eluted in the column due to the difference in retention time; subsequently, the separated components enter the ion source of mass spectrometry (MS) (e.g.,.the electron bombardment ionization source, EI), where they are ionized and fragmented under the bombardment of high-energy electrons to generate the characteristic ionic fragments; These ions are accelerated to enter the mass analyzer(e.g., quadrupole or time-of-flight analyzer), separated and detected according to the mass-to-charge ratio (m/z), and the qualitative and quantitative analysis of the compounds is finally realized by the fragmentation pattern of the mass spectra (Tranchida et al., 2018). The method has the advantages of sensitivity, low detection limit, and precision, which reduces the amount of impurities entering the chromatographic system and effectively reduces equipment contamination. However, the method requires the sample components to be sufficiently volatile and stable under the analysis condition. In the process of detection and quantification, especially alcohols, they face the problem of insufficient accuracy. Sometimes, it is found that the content may not be detected in actual experiments, or the quantitative results cannot reach the ideal conditions (Zheng et al., 2020).

In the past 5 years, researchers have carried out some improvement studies on on-line gas chromatography-mass. For example, in 2020, Zheng et al. (2020) designed a headspace-gas chromatography-mass spectrometry method to determine the ethanol content in transformer insulating oil. Applying headspace sampling techniques to DGA measurements, combined with gas chromatography and mass spectrometry, can effectively avoid many interferences. The headspace sampling technique involves placing the sample in a closed container; the sample is heated at a certain temperature for a certain period of time to equilibrate the gas and liquid phases, and the gas phase is extracted into the GC for analysis (The injection principle is shown in Figure 2B).

Online GC-MS equipment is expensive and complex to operate, making it difficult to directly apply to routine online monitoring in industrial sites. Mainly used for precise calibration of on-site online GC instruments, qualitative analysis of unknown fault gases, or handling complex fault diagnosis cases.

## 3 Spectral analysis

### 3.1 Fourier transform infrared spectroscopy

Fourier transform infrared spectroscopy (FTIR) is a spectral analysis method for differential absorption of infrared spectra due to differences in molecular structure and chemical bonding, which is an ideal on-line monitoring method for DGA analytical applications due to its high sensitivity and fast response time (Dai et al., 2025). As each chemical bond in the material only absorbs the infrared radiation at its characteristic frequency, the absorbance intensity of each light component indicates the amount of corresponding chemical bond in the examined sample. The signals measured by an FTIR spectrometer are called the FTIR spectra. It takes the form of a high dimensional vector, representing the infrared absorption intensities at a range of frequencies (Wang Z. et al., 2022). Fourier transform infrared (FTIR) spectroscopy can detect most of the DGA characteristic gases except H_2_. However, H_2_ reflects transformer partial discharge faults, which are one of the most important fault types (Fan et al., 2017). FTIR spectra are subject to multiple sources of uncertainty, and thus the analysis of them relies on domain experts and can only lead to qualitative conclusions (Tian et al., 2022). Infrared spectroscopy is easily affected by environmental factors such as humidity, which leads to inaccurate results and is limited in practical applications. In addition, Fourier transform infrared (FTIR) spectroscopy instruments are relatively large and costly to maintain, and require regular calibration and maintenance to avoid contamination (Dai et al., 2025).

**FIGURE 3:**
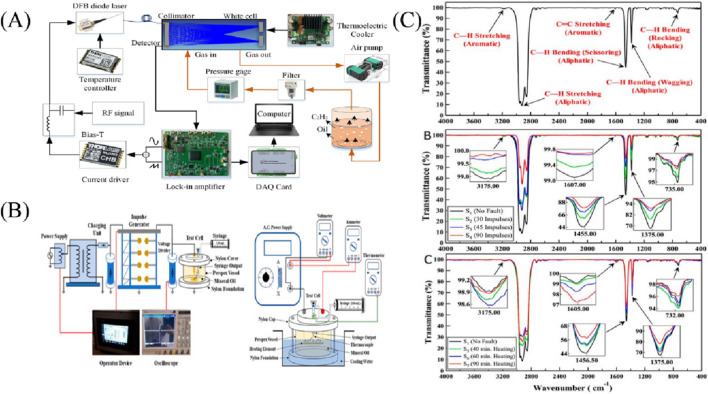
**(A)** Dissolved C_2_H_2_ sensor system in transformer oil based on FF-RF-WMS-2f/1f (Li et al., 2024); **(B)** Test cell designed by Darwish et al. (2024); **(C)** FTIR spectral data (Darwish et al., 2024).

In the last 5 years, researchers have carried out some improvement studies on FTIR. In 2019, Jiang et al. (2019a) developed an FTIR-based method for real-time accurate measurement of dissolved gases without the need for oil and gas separation. The detection limit of C_2_H_2_ was 194.5 μL/L in the 4 cm optical length range. In 2024, Darwish et al. (2024) designed two test cells (shown in Figure 3B) to monitor electrical and thermal faults in the laboratory. The use of FTIR to analyze gas samples and assess oil conditions under various fault conditions (as shown in Figure 3C) demonstrated the superiority of FTIR spectroscopic data over traditional diagnostic methods, highlighting its superior ability to detect early transformer faults. In the same year, Li et al. (2024) developed and evaluated a high-sensitivity sensor system for dissolved C_2_H_2_ in transformer oil based on fixed frequency radio frequency assisted calibration free wavelength modulation spectroscopy (FF-RF-WMS-2f/1f) (as shown in Figure 3A). By adopting a novel fixed frequency RF assisted modulation technique, the amplitude and influence of light fringes were effectively suppressed, and the equivalent MDL of dissolved C_2_H_2_ reached 0.9 nL/L.

Although FTIR has a speed advantage in multi gas synchronous detection, its inability to detect H_2_ severely limits its application as an independent device in comprehensive fault diagnosis of industrial transformers. In future industrial applications, it may be limited to specific screening scenarios that do not require H_2_ reference, or as a supplementary solution combined with other H_2_ sensors.

### 3.2 Raman spectroscopy

**FIGURE 4:**
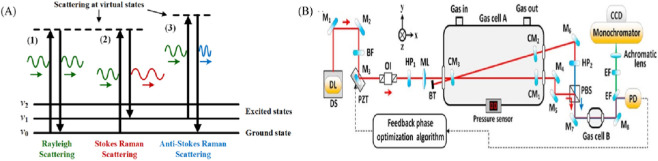
**(A)** Principle of Raman scattering (Dai et al., 2025). **(B)** Experimental setup (Wang P. et al., 2020).

Raman spectroscopy is based on the Raman scattering effect, a technique for analyzing the structure and properties of matter by measuring the Raman scattered light produced when a laser strikes a substance. The principle is that a molecule in its initial state (rotational, vibrational, or rotational-vibrational) is excited by an incident photon to a higher energy level (the virtual state or “real state” of higher electronic states), and then the molecule returns to another lower state (the final state) and emits the photonis photons (The principle is shown in Figure 4A). RS offers several advantages, including non-destructive detection, rapid analysis, simplicity, no need to separate gas mixtures, good repeatability, detection stability, and the ability to detect all gases (W et al., 2019; Ma et al., 2021). However, the Raman scattering cross section of the gas itself is small and the Raman scattering intensity of trace gases is weak, resulting in relatively low sensitivity of RS-based gas sensing.

In the last 5 years, researchers have conducted some improvement studies for Raman spectroscopy gas sensors (Dai et al., 2025). In 2020, Wang et al. designed a V-shaped cavity-enhanced Raman spectroscopy (CERS) system for identifying critical fault gases associated with transformer faults. The system utilizes an optical feedback frequency-locked technique to couple a laser into a high-precision optical resonant cavity, resulting in a significant increase in intracavity laser power, approximately 2,100 times the incident laser power. The study achieved μL/L level detection limits for nine key fault gases, including H_2_, CH_4_, and C_2_H_2_, under a total pressure of 1 bar. The study also demonstrated that extending the exposure time to 10 min can further achieve detection limits at the sub μL/L level. This work highlights the highly selective, sensitive, and accurate multi gas synchronous analysis capability of CERS technology, and its Raman signal exhibits excellent linear relationship with gas partial pressure, laser power, and exposure time, providing a powerful solution for transformer fault diagnosis. (The experimental setup is shown in Figure 4B) (Wang P. et al., 2020). In 2022, Wang et al. developed a fiber-enhanced RS system for the detection of dissolved gases using a hollow anti-resonant fiber. This system was responsible for detecting various gases in the air around the laboratory (Wang J. et al., 2022). It achieved a stabilized laser-fiber coupling efficiency of about 85%. The MDLs of H_2_, CO, CO_2_, CH_4_, C_2_H_6_, C_2_H_4_ and C_2_H_2_ gases were 8, 22, 5, 2, 6, 3 and 3 ppm, respectively.

RS is difficult to meet the detection requirements of transformer fault gases at the μL/L/ppb level, and is basically in the research stage. Mature industrial grade online monitoring products have not yet been developed.

### 3.3 Photoacoustic spectrometry

Photoacoustic Spectroscopy (PAS) is an important gas detection technique that utilizes the photoacoustic thermal effect to analyze the composition of dissolved gases in transformer oil by measuring the pressure wave (frequency range 10 Hz–100 kHz) generated by the absorption of modulated light (wavelength range from mid-infrared to terahertz) to detect the gas. Compared with the traditional absorption spectroscopy, fluorescence spectroscopy or Raman spectroscopy techniques, the PAS technique is generally less affected by light scattering and typically offers a near-zero background with strong anti-interference capability, which has the advantages of zero background and strong anti-interference. At the same time, It also has the advantages of wavelength-independent detectors and a relatively simple structure, and is capable of real-time *in situ* monitoring due to its fast response time, compact structure, and high sensitivity (Wei et al., 2024). As a non-destructive testing method, photoacoustic spectroscopy (PAS) technology also has potential for on-line detection of trace dissolved gases (Zhang, 2025). Therefore, PAS has also become one of the ideal online monitoring methods for DGA analysis applications. Gas sensors based on Photoacoustic Spectroscopy (PAS) have the advantages of high sensitivity, wide dynamic range, low cost, and small footprint, making them ideal for energy, environment, safety, and public health applications. Ideal for many fields (Paris et al., 2024). However, multi-gas sensing in PAS usually requires the use of multiple lasers, each targeting the specific absorption properties of different gases, leading to increased system complexity, high cost, and maintenance requirements.

**FIGURE 5:**
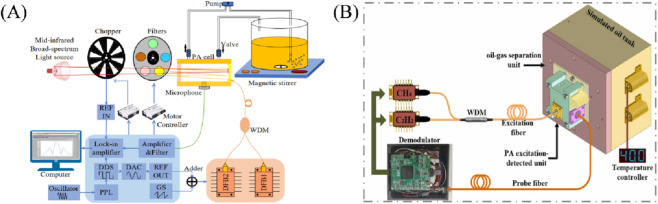
**(A)** Schematic diagram of the MPAEPAS experimental setup (Li et al., 2022); **(B)** Schematic diagram of the *in-situ* detection system for dissolved C_2_H_2_/CH_4_ gases (Li et al., 2025).

In the last 5 years, researchers have conducted several improvement studies on PAS. For example, in 2019, Zhou and Iannuzzi (2019) designed an immersion PAS (iPAS) system specifically for detecting arc faults. This iPAS system can be directly immersed in an oil bath to detect dissolved C_2_H_2_ with a minimum detection limit of 47 nL/L for C_2_H_2_. The system does not require oil/gas separation, has a simple structure. In 2020, Yang et al. (2020) used a fiber laser power amplification technique to increase the laser power from 20 mW to 200 mW. They developed a cantilever beam Fabry-Pérot (F-P) fiber optic acoustic sensor probe designed to match the resonant frequency of the photoacoustic unit. This configuration achieves a dual resonance enhancement of the cantilever beam and the photoacoustic unit. The system achieved an MDL of 15 nL/L for C_2_H_2_. In 2021, Sun and Cao (2021) developed an online detection device for dissolved gases in transformer oil based on photoacoustic spectroscopy technology. By optimizing the headspace degassing and multi band filtering system, combined with high-sensitivity microphones and dedicated H_2_ sensors, high-precision synchronous detection of H_2_, CO_2_, CO_2_ and various hydrocarbon gases was achieved. The detection limits for C_2_H_2_ and H_2_ were 0.5 ppm and 5.0 ppm, respectively. In the same year, Chen T. et al. (2021) introduced a portable PAS system that utilized an erbium-doped fiber amplifier (EDFA) to further improve the detection sensitivity. A C_2_H_2_ detection limit of 3.4 ppb was achieved. In the same year, Chen K. et al. (2021) developed a high-sensitivity fiber optic sensor based on photoacoustic spectroscopy (PAS) technology, which achieves *in-situ* monitoring of dissolved gases in oil by detecting the sound waves generated by gas absorption of laser. At 50 °C, as the temperature increases, the equilibrium time for oil and gas separation shortens,and temperature compensation greatly improves measurement accuracy. This provides a detection solution for transformer fault diagnosis that is free of chromatographic separation, resistant to electromagnetic interference, and can be deployed *in situ* for a long time. This provides a detection solution for transformer fault diagnosis that is free of chromatographic separation, resistant to electromagnetic interference, and can be deployed *in situ* for a long time. 2022, Chen et al. (2022) developed a high-sensitivity detection method that combines fiber optic sensing and photoacoustic spectroscopy principles, successfully achieving specificity, anti-interference, and rapid detection of extremely low concentrations of acetylene dissolved in transformer oil (up to 10 nL/L or lower), providing a powerful potential technical means for power equipment state monitoring. In the same year, Li et al. (2022) proposed a multi-channel absorption-enhanced photoacoustic spectrometer (MPAEPAS) based on the analytical dissolution of a combined light source (e.g., Figure 5A). Absorption enhancement was achieved by multiple reflections, resulting in detection limits of 30, 100, 3, 0.5, 3, and 3 μL/L for the detection of dissolved CO, CO_2_, CH_4_, C_2_H_2_, C_2_H_4_, and C_2_H_6_, respectively. The instrument was highly accurate and provided a technological solution for the monitoring of dissolved multicomponent gases. In 2024, Xu et al. (2025), based on the C_1_, C_2_, and C_3_ gases infrared absorption spectra, analyze the reasons for gas detection interference and design a C_3_ high hydrocarbon gas filtering device to reliably separate the dissolved C_3_ high hydrocarbon gases in the oil, eliminate the interference of the low hydrocarbon gases of C_1_ and C_2_ to be measured, and improve the accuracy of CH_4_, C_2_H_6_, and C_2_H_4_ gas detection, which helps to improve the operational safety and reliability of the transformer. In 2025, Li et al. (2025) developed a frequency division multiplexing fiber optic photoacoustic sensor, which achieved *in-situ* synchronous detection of dissolved H_2_/CH_4_ in transformer oil based on photoacoustic spectroscopy (PAS) technology (Figure 5B is a schematic diagram of the detection system), providing real-time and high-precision diagnostic tools for early faults in transformers such as local overheating and discharge.

PAS is one of the technologies with the greatest potential to challenge the position of online GC at the industrial level. It does not require a carrier gas and separation column, has low maintenance requirements, and high sensitivity, which is very suitable for the dual needs of reliability and cost-effectiveness in industrial sites. At present, PAS technology is rapidly moving from laboratory research and development to commercialization, and multiple manufacturers have launched industrial products.

### 3.4 Tunable diode laser absorption spectroscopy

**FIGURE 6:**
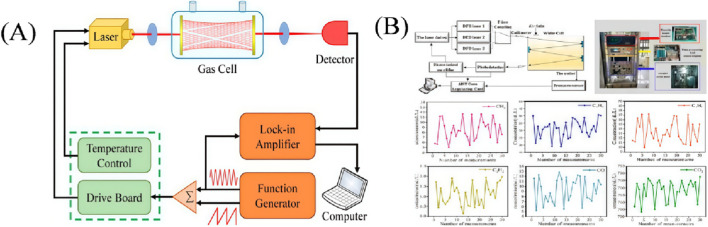
**(A)** Schematic diagram of TDLAS for analyzing DGA (Dai et al., 2025); **(B)** Methodological diagram of TDLAS for analyzing DGA (Chen Y. et al., 2021).

Tunable Diode Laser Absorption Spectroscopy (TDLAS) is a spectroscopic analysis method that uses a narrow band laser to scan molecular absorption spectra and then obtains the parameters of the gas to be measured by analyzing the intensity of the laser light absorbed by the gas molecules (the analytical principle is shown in Figure 6A). TDLAS, which is characterized by good selectivity, high sensitivity, fast response time, and high interference immunity, is able to accurately detect trace gases in a very efficient manner and to achieve noncontact on-line measurement (Zhou et al., 2019). The second harmonic and first harmonic label-free method modulates the target signal at high frequency during the measurement process, while the non-target signal is removed in the subsequent harmonic detection process due to the lack of modulation; at the same time, the use of the first harmonic to the second harmonic normalization eliminates the effect of light intensity, which can effectively reduce the noise signal interference in the measurement system, with the advantage of a high signal-to-noise ratio; solves the problem of the multi-channel gas cell volume caused by the headspace gas volume dilution problem, which improves the minimum detection limit (MDL) of dissolved gases. These advantages make this method one of the ideal online monitoring methods for DGA analysis applications. However, in multi-gas sensing applications, TDLAS usually requires multiple lasers to target the different absorption characteristics of each gas, which can complicate system design and increase operating costs (Sun and Cao, 2021; Zhou et al., 2019). TDLAS consists of both direct absorption spectroscopy and wavelength-modulated spectroscopy methods. Among them, the direct absorption method uses the light intensity in the absorption-free region as a baseline for the measurement of the gas absorbance function, but this method is susceptible to factors such as particulate concentration, laser intensity fluctuation, and baseline fitting uncertainty in the measurement (Zhou et al., 2019).

In the last 5 years, researchers have conducted some improvement studies on TDLAS (Dai et al., 2025). In 2019, Jiang et al. (2019b) introduced a TDLAS system for multi-gas sensing. The system utilized a gas cell with an optical length of 10.13 m to further mitigate the effects of field vibrations. Acetylene detection reaches one millionth, while other hydrocarbon gases reach μL/L levels. TDLAS-based online test equipment was deployed for monitoring 220 kV main transformers, and the error level of the system met the criteria of the relevant standards. In 2021, Chen Y. et al. (2021) developed an online sensing system using TDLAS to measure the dissolved multicomponent concentrations of gases. The system uses three lasers to detect the gases: a 1,684 nm laser for CH_4_, C_2_H_4_, and C_2_H_6_; a 1,579 nm laser for CO and CO_2_; and a 1,532 nm laser for C_2_H_2_. The experimental setup is shown in Figure 6B. The experimental results show that C_2_H_2_ is detected with an error of nL/L level, while the other gases are detected with errors in the μL/L range, and that the experimental results comply with the test criteria and can be used to assess the condition of the transformer (Dai et al., 2025).

TDLAS has been successfully applied in industry for ultra-high sensitivity and rapid response specialized monitoring of specific key gases such as C_2_H_2_ and H_2_. However, due to the need to integrate multiple lasers for the full component analysis of C_1_- C_2_, the system complexity and cost have sharply increased, limiting its industrial promotion as a universal comprehensive DGA solution. But it can still serve as a powerful supplement to GC or PAS systems, providing early and accurate warnings for specific types of faults.

## 4 Gas sensor methods

The gas sensor is a device that converts gas concentration into an electrical signal, with a gas-sensitive material at its core. When used for insulating oil dissolved gas detection, the characteristic faulty gas in the oil is separated by a permeable membrane, and the physical/chemical interaction between the gas and the sensitive material causes changes in electrical characteristics such as resistance and capacitance, and outputs a signal. Advantages include high sensitivity (μL/L level), fast response, miniaturization and integration, and no need for complex degassing; disadvantages include lack of selectivity due to cross-talk of mixed gases, fluctuating oil temperatures affecting stability, and long-term oil immersion triggering material deterioration requiring frequent calibration. In the last 5 years, researchers have conducted some improvement studies for gas sensors.

### 4.1 C_2_H_2_

**FIGURE 7:**
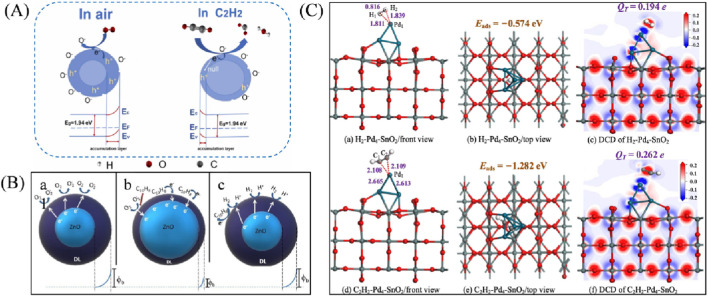
**(A)** Schematic of the sensing mechanism (Tang et al., 2025); **(B)** Schematic explanation of the adsorption interaction on the sensor surface with depletion layer (DL) and potential barrier: a. In an air atmosphere, b. After immersion in mineral oil, and c. Adsorption of H_2_ molecules (Lustosa et al., 2025); **(C)** H_2_ and C_2_H_2_ adsorption on Pd_4_-SnO_2_ and DCD analysis (Liu et al., 2022).

In 2019, Gui et al. (2020) used antimony-doped graphene gas-sensitive material (Sb-CT) as an adsorption substrate, and established a structural model and gas molecule adsorption model of graphene-based gas-sensitive material based on density-functional theory (DFT), and found that gas molecules can be desorbed from the surface of graphene material after absorbing a certain amount of heat, which makes the graphene-based sensors reusable in detecting dissolved gases in oils. But antimony-doped graphene is more sensitive to acetylene (C_2_H_2_) and ethylene (C_2_H_4_), less sensitive to hydrogen (H_2_). In 2020, Wang Y. et al. (2020) designed an optical gas sensing with hollow photonic crystal fiber (HC-PCF) as the absorbing gas cell, which has no cross-talk and high sensitivity for detection of acetylene (C_2_H_2_), with an average time of 29 s and a minimum detection limit of up to 4.5 μL/L. In 2025, Tang et al. (2025) synthesized a SmFeO_3_ rich in surface oxygen vacancies using lyophilization-assisted sol-gel method and achieved a detection limit of 200 nL/L for acetylene. The sensor has a low detection limit and high stability, in addition to high selectivity for acetylene compared to other gases (C_2_H_4_, CH_4_, NO, CO, H_2_). (Schematic of the sensing mechanism is shown in Figure 7A).

### 4.2 C_2_H_6_

In 2020, Chang et al. (2024) developed a new zinc oxide (ZnO) thin-film ethane gas sensor, which uses the metal oxide zinc oxide as an n-type semiconductor for gas detection, “Metal Oxide Mechanism”. This sensor not only has a high gas-sensitive response capability, which can realize a significant resistance change to ethane gas in a short period of time, but also has a good reproducibility, which maintains a stable response characteristic over different test cycles.

### 4.3 CO

2023, Morandi Lustosa et al. (Simões et al., 2024) developed a monolayer SnO_2_ sensor based on Pd nanoparticle modification for real-time detection of CO dissolved gas in transformer oil, which was investigated by monitoring the change in conductivity for the detection of CO dissolved in mineral oil. Its detection limit for CO was as low as 13.3 μL/L even at very low concentrations. In the same year, Xie et al. (2024a) developed a high-performance room temperature CO gas sensor based on CuO/TiO_2_/N-MWCNTs ternary nanocomposites. The sensor is suitable for detecting trace amounts of CO gas dissolved in transformer oil and provides a key means for online monitoring and early warning of internal overheating faults in transformers. In 2024, Xie et al. (2024b) developed MOF-derived NiO/SnO_2_ prepared using a hydrothermal method as a composite-based sensor for the efficient detection of CO gas at room temperature. The sensor exhibited excellent response/recovery characteristics, selectivity and long-term stability with very high response (5.48@100 μL/L CO gas) and very large detection limit (1–100 μL/L) for CO gas at room temperature. In 2024, Huang et al. (2024) developed a gas sensor material based on metal clusters (Pth_3_, Rhh_3_) modified WTe_2_ monolayers. The sensor achieved high sensitivity and selectivity for detecting fault characteristic gases (CO, CO_2_, H_2_, C_2_H_2_) dissolved in transformer oil at room temperature, with Rhh_3_ WTe_2_ exhibiting specific response to CO and CO_2_ (suitable for resistive sensors).

### 4.4 H_2_

In 2025, Agnes Nascimento Simões et al. (Lustosa et al., 2025) developed a gas sensor based on nano engineering PDMS/Pd/ZnO composite structure, which achieves high sensitivity and selectivity in detecting hydrogen gas (H_2_) dissolved in transformer oil at room temperature, significantly improving the convenience and energy efficiency of hydrogen gas analysis in oil. Figure 7B is Adsorption interaction on sensor surface and depletion layer (DL) and potential barrier. And Pt_3_-WTe_2_ can distinguish four gases through changes in work function, solving the problem of poor selectivity and high temperature dependence of traditional sensors in complex gas environments in oil.

### 4.5 H_2_, C_2_H_2_

In 2022, Liu et al. (2022) significantly improved the detection performance of key fault gases (H_2_ and C_2_H_2_) in oil immersed transformers by developing SnO_2_ nanowire gas sensors modified with Pd_4_ clusters. Exhibiting high sensitivity and excellent selectivity. This achievement effectively solves the problem of insufficient sensitivity and cross sensitivity of traditional metal oxide semiconductor sensors to trace fault gases, providing a reliable and efficient technical solution for intelligent online monitoring of fault gases in power equipment (H_2_ and C_2_H_2_ adsorption on Pd_4_-SnO_2_ and DCD analysis as shown in Figure 7C).

Gas sensors based on metal oxides or new materials are an ideal choice for low-cost, distributed monitoring of single gas (such as H_2_). In order to adapt to DGA analysis, array type gas sensors should be developed in the future for simultaneous and high-sensitivity sensing of multiple gases.

## 5 Summary and outlook

Online detection technology enables continuous acquisition of transformer operational status information, helping maintenance personnel promptly monitor equipment health. Should potential fault indicators emerge, staff can immediately receive early warnings and swiftly implement maintenance measures. This not only significantly enhances transformer operational reliability but also substantially reduces lifecycle management costs for power equipment (Youssef et al., 2025).

Measured against GC, the industry gold standard, emerging monitoring technologies have achieved significant breakthroughs in multiple key performance metrics. Taking photoacoustic spectroscopy (PAS) as an example, its detection limit has been elevated to the nL/L level, matching GC performance; it can simultaneously detect multiple gas components including CO, CO_2_, and C_1_–C_2_ hydrocarbons, while demonstrating excellent resistance to electromagnetic interference, revealing its potential to directly compete with GC. However, this technology still relies on periodic GC calibration for long-term operational stability and quantitative accuracy, and its practical reliability requires further validation through extensive field-based long-term operational data. Tunable Diode Laser Absorption Spectroscopy (TDLAS) has pursued a differentiated development path. Rather than striving for comprehensive gas coverage, it focuses on maximizing detection performance for specific critical gases (e.g., C_2_H_2_, H_2_). In response speed and single-gas sensitivity, it even surpasses GC. Therefore, its performance evaluation should emphasize specialized capabilities in specific fault warning scenarios, rather than aiming for comprehensive replacement of GC. Gas sensor technology, meanwhile, makes a clear trade-off between performance and cost. Its detection limits currently range at the μL/L level, and it still falls far short of GC in selectivity and long-term stability. However, its core value lies in achieving extreme optimization in cost, size, and power consumption. This makes it suitable for large-scale, distributed trend monitoring scenarios where GC is impractical, providing foundational technical support for building condition monitoring networks.

However, transitioning current online monitoring technologies from laboratory research to large-scale industrial application still faces significant challenges. While various monitoring techniques perform well in controlled laboratory settings, their long-term stability and reliability in the harsh environments of actual substations remain to be verified. Key challenges manifest in three areas: 1. Insufficient adaptability to complex environments. Outdoor monitoring equipment must endure significant temperature fluctuations, which directly impact sensor baseline stability and oil-gas separation efficiency. Additionally, high humidity environments can cause condensation on optical components, not only degrading the optical performance of systems like FTIR and PAS but also potentially triggering circuit board short circuits. 2. Stringent electromagnetic compatibility requirements. Intense electromagnetic interference sources within substations significantly impact precision measurement units such as gas sensors and chromatographic detectors. This leads to measurement data distortions like drift, abnormal spikes, or persistent repetition, severely compromising the accuracy of fault diagnosis. 3. Conflict between system durability and maintenance. Online monitoring systems require long-term maintenance-free operation. However, in practice, GC columns gradually degrade and fail, optical detection windows become contaminated, and gas-sensitive materials in oil-immersed environments experience performance decay. These factors necessitate frequent system calibration, making it difficult to ensure long-term measurement reliability.

Furthermore, existing systems suffer from inherent limitations: limited detection of characteristic gases results in insufficient diagnostic evidence; monitoring software offers limited functionality, failing to meet intelligent diagnostic demands; prolonged operation in harsh environments frequently causes data anomalies or gaps, directly compromising fault analysis accuracy. These bottlenecks severely constrain the deep application of online DGA technology in condition-based maintenance (He et al., 2024).

To overcome existing technical bottlenecks and meet the higher demands of smart grids for equipment condition monitoring, online DGA technology must achieve breakthroughs in the following key areas: 1. Simultaneous detection of multiple gas components. It is essential to overcome the limitations of current single-gas or limited-gas detection methods. Focus should be placed on developing multispectral sensor arrays and data fusion technologies to enable simultaneous analysis of nine characteristic gases, including C_1_-C_3_ hydrocarbons. This comprehensive coverage of gas components is fundamental for accurately determining fault types and significantly reducing misdiagnosis rates, driving fault diagnosis from partial judgments toward systematic evaluations. 2. System miniaturization and cost reduction. Current technical solutions reliant on precision optical components, chromatographic columns, and other core parts face challenges of high costs and frequent maintenance, hindering large-scale deployment. Future development should concentrate on creating simpler, more economical monitoring methods. Examples include micro-sensor arrays based on novel sensitive materials (such as MOFs and two-dimensional materials) and robust optical platforms capable of maintaining high sensitivity within simplified structures. This approach will substantially reduce system costs and maintenance requirements while ensuring performance. 3. Intelligent Upgrades and Predictive Capabilities. Next-generation online DGA systems must transcend basic data collection by deeply integrating artificial intelligence and digital twin technologies. By incorporating machine learning algorithms, systems can extract equipment health information from gas concentration data, enabling a strategic shift from “scheduled maintenance” to “predictive condition-based maintenance.” This transformation will comprehensively enhance the precision of power asset management and improve grid operational reliability. 4. Enhanced Robustness and Environmental Adaptability: Acknowledging the harsh realities of substation environments is paramount for successful industrial adoption. Future systems must be purposefully designed for long-term stability and reliability. Key research directions include: (1) Hardware Robustness: Developing sensors and optical components with inherent resistance to electromagnetic interference (EMI), wide temperature fluctuations, and high humidity. (2) Data Reliability Algorithms: Creating intelligent algorithms capable of identifying and correcting for data anomalies caused by environmental interference, such as signal drift, spikes, and persistent repeated values. These self-diagnosing and self-correcting capabilities are essential for generating trustworthy data. (3) Maintenance-free Operation: Advancing materials and designs that mitigate column aging in GC, optical window fouling in spectroscopic systems, and sensor degradation in oil, thereby extending calibration cycles and achieving truly unattended operation.

In conclusion, the most promising path forward for industrial DGA lies in simultaneously advancing multi-component PAS systems and strategically adopting hybrid monitoring architectures. This requires combining the baseline robustness of GC calibration with the real-time, targeted tracking capabilities of optical technologies such as PAS and TDLAS. This collaborative framework provides the optimal balance of accuracy, reliability, and cost, which is crucial for accelerating the full integration of intelligent DGA into the digital grid and building a more dynamic and sustainable power infrastructure.
